# Incremental Values of T1 Mapping in the Prediction of Sudden Cardiac Death Risk in Hypertrophic Cardiomyopathy: A Comparison With Two Guidelines

**DOI:** 10.3389/fcvm.2021.661673

**Published:** 2021-06-08

**Authors:** Le Qin, Jiehua Min, Chihua Chen, Lan Zhu, Shengjia Gu, Mi Zhou, Wenjie Yang, Fuhua Yan

**Affiliations:** ^1^Department of Radiology, Ruijin Hospital, Shanghai Jiao Tong University School of Medicine, Shanghai, China; ^2^Department of Cardiac Surgery, Ruijin Hospital, Shanghai Jiao Tong University School of Medicine, Shanghai, China

**Keywords:** hypertrophic cardiomyopathy, sudden cardiac death, magnetic resonance imaging, native T1 mapping, ECV, guideline

## Abstract

**Background:** MRI native T1 mapping and extracellular volume fraction (ECV) are quantitative values that could reflect various myocardial tissue characterization. The role of these parameters in predicting the risk of sudden cardiac death (SCD) in hypertrophic cardiomyopathy (HCM) is still poorly understood.

**Aim:** This study aims to investigate the ability of native T1 mapping and ECV values to predict major adverse cardiovascular events (MACE) in HCM, and its incremental values over the 2014 European Society of Cardiology (ESC) and enhanced American College of Cardiology/American Heart Association (ACC/AHA) guidelines.

**Methods:** Between July 2016 and October 2020, HCM patients and healthy individuals with sex and age matched who underwent cardiac MRI were prospectively enrolled. The native T1 and ECV parameters were measured. The SCD risk was evaluated by the 2014 ESC guidelines and enhanced ACC/AHA guidelines. MACE included cardiac death, transplantation, heart failure admission, and implantable cardioverter-defibrillator implantation.

**Results:** A total of 203 HCM patients (54.2 ± 14.9 years) and 101 healthy individuals (53.2 ± 14.7 years) were evaluated. During a median follow-up of 15 months, 25 patients (12.3%) had MACE. In multivariate Cox regression analysis, global native T1 mapping (hazard ratio (HR): 1.446; 95% confidence interval (CI): 1.195–1.749; *P* < 0.001) and non-sustained ventricular tachycardia (NSVT) (HR: 4.949; 95% CI, 2.033–12.047; *P* < 0.001) were independently associated with MACE. Ten of 86 patients (11.6%) with low SCD risk assessed by the two guidelines had MACE. In this subgroup of patients, multivariate Cox regression analysis showed that global native T1 mapping was independently associated with MACE (HR: 1.532; 95% CI: 1.221–1.922; *P* < 0.001). In 85 patients with conflicting results assessed by the two guidelines, end-stage systolic dysfunction was independently associated with MACE (HR: 7.942, 95% CI: 1.322–47.707, *P* = 0.023). In 32 patients with high SCD risk assessed by the two guidelines, NSVT was independently associated with MACE (HR: 9.779, 95% CI: 1.953–48.964, *P* = 0.006).

**Conclusion:** The global native T1 mapping could provide incremental values and serve as potential supplements to the current guidelines in the prediction of MACE.

## Introduction

Hypertrophic cardiomyopathy (HCM) is a common genetic disease with a prevalence of 1/200 to 500 people (1–3). Although a majority of HCM patients have normal life expectancy, sudden cardiac death (SCD) remains to be the most common adverse outcome (4). Implantable cardioverter-defibrillator (ICD) is recommended as the only effective way to prevent from SCD and increase lifespan (5). However, placement of ICDs is sometimes companied by complications such as infections (6). Thus, it is important but challenging to identify patients who are at high risk of SCD and likely benefit from ICDs.

Two strategies are widely used for assessing SCD risk in HCM patients in clinical settings, which often leads to conflicting results (7). One is the 2014 European Society of Cardiology (ESC) guidelines in which a 5-year SCD risk predictive model was proposed (8), and the other is the enhanced American College of Cardiology/American Heart Association (ACC/AHA) guidelines that included seven binary biomarkers to judge SCD risk (9). In the enhanced ACC/AHA guidelines, the percentage of late gadolinium enhancement (LGE) to left ventricular (LV) mass representing the myocardial characterization is included as a new biomarker. This feature makes the two guidelines very different. LGE could be non-invasively acquired by cardiac MRI and has been a reference standard for evaluating myocardial fibrosis (10). In the past decade, LGE has been confirmed as an independent predictor of SCD and adverse outcomes in HCM (11–13). However, it is often confusing to define diffuse interstitial expansion by LGE (14). The amount of LGE also differs based on the chosen thresholding techniques (15).

MRI mapping technique can non-invasively reflect tissue physiology and pathophysiology in multiple cardiovascular diseases alternative to myocardial biopsies (16). Native T1 mapping and extracellular volume fraction (ECV) derived from native and contrast-enhanced T1 values are quantitative methods that enable the detection of myocardial tissue characterization (17). These intrinsic quantitative parameters can reflect the states of focal and diffuse interstitial fibrosis, as well as iron overload, lipid deposition, edema, and protein infiltration (18–20). Compared with LGE, native T1 mapping and ECV could potentially provide more information about myocardial tissue. However, the predictive value of these parameters has not been fully understood.

Hence, this study was conducted to evaluate the ability of native T1 and ECV values to predict major adverse cardiovascular events (MACE) in HCM patients, and its incremental values over the current 2014 ESC guidelines and enhanced ACC/AHA guidelines.

## Materials and Methods

**Figure 1:**
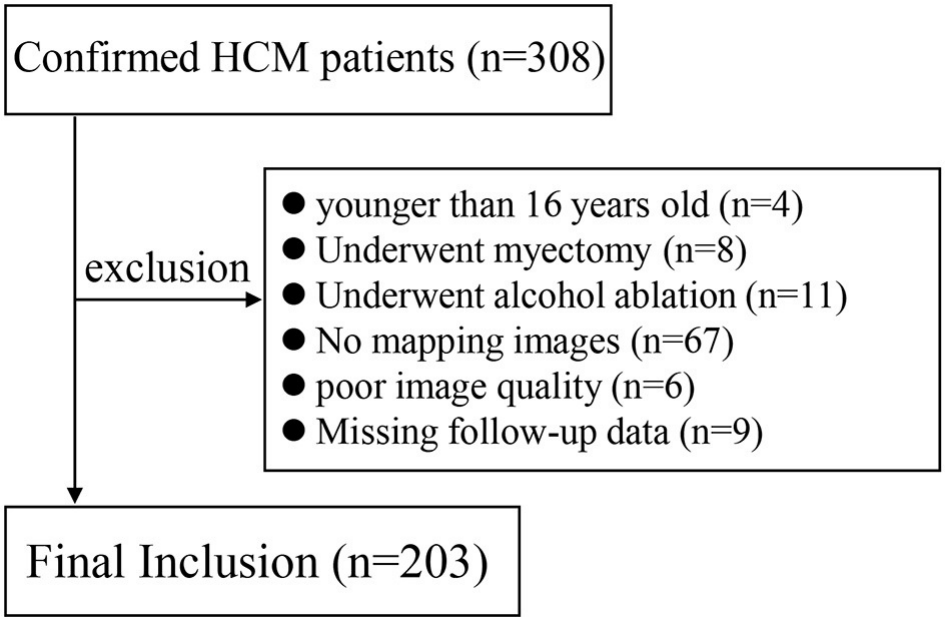
Flow diagram of patient selection.

This prospective study was approved by our local Institutional Review Board, and written informed consents of all patients were obtained. From July 2016 to October 2020, 308 HCM patients who underwent cardiac MRI in our institution were enrolled. Clinical diagnosis of HCM was done based on the LV wall thickness (LVWT) ≥15 mm by echocardiography or MRI without LV dilation that could not be explained by loading conditions or LVWT of 13–14 mm supported by family history of HCM, non-cardiac symptoms, electrocardiogram, laboratory tests, and other cardiac imaging (1, 8). Differentiation from hypertensive heart diseases were summarized in Supplementary Material 1. Exclusion criteria are shown in Figure 1. Follow-up data was acquired by telephone interview, outpatient visit, and the record of inpatient data. The primary endpoint was set as the incidence of MACE, including all cardiac-caused death, the placement of ICDs, cardiac transplantation, myocardial infarction, and heart failure hospitalization. One hundred one healthy volunteers with age- and sex-matched distribution with HCM patients were enrolled as control group, with no history of cardiovascular diseases and symptoms, and with normal electrocardiogram and echocardiography. The control group was included to define the normal ranges of native T1 mapping and ECV values.

### Cardiac MRI Protocols

All cardiac MRI examinations were performed on a 3.0-T MRI scanner (Ingenia, Philips Healthcare). The cine (voxel: 2 × 2 × 8 mm, field of view: 350 × 350 × 8 mm, time repetition/time echo: 3.1/1.55 ms, flip angle: 45°, matrix: 176 × 168, sense factor: 2) and LGE (voxel: 1.6 × 1.9 × 10 mm, field of view: 300 × 300 × 10 mm, time repetition/time echo: 6.1/3.0 ms, flip angle: 25°, matrix: 188 × 135, sense factor: 2) sequences included two-, three-, and four-chamber and short-axis images covering the whole LV myocardium. Native and enhanced T1 mapping were acquired by MOdified Look-Locker recovery sequence using “5s(3s)3s” and “4s(1s)3s(1s)2s” scheme, respectively (voxel: 2 × 2 × 8 mm, FOV: 300 × 300 × 8 mm, TR/TE: 2.3/1.07 ms, flip angle: 20°, matrix: 152 × 150) (Supplementary Material 2). Mapping image acquisition was in short-axis slices with a gap of 2 mm covering the whole LV myocardium. LGE and enhanced T1 mapping were performed 10–15 min after intravenous administration of gadolinium contrast agent (Magnevist, Bayer Healthcare Pharmaceuticals) of 0.2 mmol/kg.

### MRI Analysis

Cardiac structure, function, and LGE imaging were analyzed on a commercial postprocessing software QMass (version8.1, Medis Medical Imaging). Phenotypes of HCM were evaluated on four-chamber cine images as previously described (4). The maximal LVWT was measured on cine images of all planes at the end-diastolic phase. The anterior-posterior left atrium (LA) diameter was measured on four-chamber cine images. Cardiac function was calculated by manually tracing the endocardial and epicardial borders of LV myocardium at the end-diastolic and end-systolic phases on short-axis cine images, excluding papillary muscles. The quantitative LGE was performed by manually adjusting the grayscale intensity threshold slider to fill in the visually apparent hyperintense as described previously (21). Global and regional native T1 mapping and ECV values were acquired on a commercial post-processing software (CVI^42^, Circle Cardiovascular Imaging) by manually tracing endocardial and epicardial borders of LV myocardium on motion-corrected mapping images. The reference points were set at the superior and inferior LV insertion to generate a 16-segment AHA model (22). Hematocrit was recorded by obtaining venous blood sample within 24 h prior to MRI. The ECV values were generated by equations reported previously (18). The minimal and maximal mapping values of all 16 segments were also selected for analysis.

### Risk Models

The family history of SCD and unexplained syncope were recorded. The standard echocardiography was used to measure an instantaneous peak Doppler LV outflow tract (LVOT) pressure gradient at rest (8). The 24-h ambulatory electrocardiogram monitoring was performed to detect the presence of non-sustained ventricular tachycardia (NSVT) in a standard fashion (5, 8).

A 5-year estimated SCD risk was calculated according to the formulation of “1-0.998^exp(Prognostic index)^” proposed in the 2014 ESC guidelines (8). Prognostic index = [0.15939858 × maximal LVWT (mm)]–[0.00294271 × maximal LVWT^2^ (mm^2^)] + [0.0259082 × left atrial diameter (mm)] + [0.00446131 × maximal (rest/Valsalva) LVOT gradient (mmHg)] + [0.4583082 × family history of SCD] + [0.82639195 × NSVT] + [0.71650361 × unexplained syncope]–[0.01799934 × age at clinical evaluation (years)]. Patients with 5-year risk of <4 and ≥4% were classified as lower and higher SCD risk groups, respectively. According to the enhanced ACC/AHA guidelines, patients were considered at high risk for SCD if at least one of the following major risk markers was present: family history of SCD, maximal LVWT ≥30 mm, unexplained syncope, NSVT, LGE/LV mass ≥15%, end-stage LV ejection fraction (LVEF) <50%, and LV apical aneurysm (9).

### Statistical Analysis

All continuous data were expressed as mean ± standard deviation if normally distributed, and median (interquartile range) if non-normally distributed. The normality of data was determined by Kolmogorov-Smirnov test. Independent *t*-test and Mann-Whitney *U*-test were used to compare the normally and non-normally distributed data between two groups, respectively. The ordinal variables were compared by χ^2^ test or Fisher's exact test, when appropriate. ANOVA test and Kruskal–Wallis *H*-test were used to compare the normally and non-normally distributed data among three groups, respectively, with Bonferroni test for paired *post-hoc* test. Univariate and multivariate Cox regression analyses were used to estimate the relationship between cardiac MRI and major SCD risk, with the incidence of MACE. “Forward: LR” was used to select variables in the multivariate analysis. Kaplan-Meier methods and log-rank tests were used to compare the MACE-free survival between two groups. Pearson (*r*) and Spearman (*r*_*s*_) correlation coefficient were used to analyze the correlation of continuous data and ranking data, respectively. All statistical analyses were performed by SPSS (version22.0, IBM) and GraphPad Prism (version8.0.2, GraphPad Software). *P* < 0.05 was considered to be statistically significant.

## Results

### Study Population

A total of 203 HCM patients (120 males, 59.1%; age, 54.2 ± 14.9 years) and 101 healthy individuals (59 males, 58.4%; age, 53.2 ± 14.7 years) were included. Eighty-six patients (42.4%) were older than 60 years. Hypertension was common in 126 HCM patients (62.1%), and LVOT pressure gradient of 30 mmHg or greater was present in 105 patients (51.7%). Thirteen patients had SCD family history, 28 underwent unexplained syncope, and 11 had NSVT. All baseline demographics are summarized in Table 1.

**Table 1:** Baseline demographics of HCM patients and healthy individuals.

**Baseline demographics**	**HCM patients (*N* = 203)**	**Control group (*N* = 101)**	***P*-value**
**Clinical data**			
Male (*N*, %)	120, 59.1%	59, 58.4%	0.907
Age (years)^+^	54.2 ± 14.9	53.2 ± 14.7	0.592
BMI (kg/m^2^)^+^	24.8 ± 3.7	23.5 ± 2.5	<0.001^*^
Heart rate (beats/min)^#^	70 (18)	67 (15)	0.001^*^
Systolic blood pressure (mmHg)^#^	132 (27)	117 (15)	<0.001^*^
Diastolic blood pressure (mmHg)^#^	74 (14)	71 (11)	0.043
Hypertension (*N*, %)	126, 62.1%	0, 0%	<0.001^*^
Hyperlipidemia (*N*, %)	59, 29.1%	0, 0%	<0.001^*^
Diabetes mellitus (*N*, %)	24, 11.8%	0, 0%	<0.001^*^
Atrial fibrillation (*N*, %)	22, 10.8%	0, 0%	<0.001^*^
SCD family history (*N*, %)	13, 6.4%	0, 0%	0.009^*^
Unexplained syncope (*N*, %)	28, 13.8%	0, 0%	<0.001^*^
NSVT (*N*, %)	11, 5.4%	0, 0%	0.017^*^
Mitral regurgitation	157, 77.3%	0, 0%	<0.001^*^
NYHA classification (I/II/III/IV)	18/82/100/3	101/0/0/0	<0.001^*^
**Echocardiography**			
LOVT pressure gradient (mmHg)^#^	32 (49)	–	–
**Laboratory test**			
NT-pro BNP (pg/ml)	1,216.4 (1,196.1)	–	–
CK-MB (ng/ml)	3.5 (2.7)	–	–
Myoglobin (ng/ml)	21.6 (11.5)	–	–
cTnI (ng/ml)	0.03 (0.06)	–	–
**Therapy**			
Septal myectomy (*N*, %)	99, 48.8%	–	–
ICD (*N*, %)	8, 3.9%		
**Drug therapy**^**$**^			
β-Blocker (*N*, %)	87, 42.9%	–	–
ACEi/ARB (*N*, %)	92, 45.3%	–	–
Calcium antagonists (*N*, %)	62, 30.5%	–	–
Diuretics (*N*, %)	9, 0.4%	–	–

*HCM, hypertrophic cardiomyopathy; BMI, body mass index; SCD, sudden cardiac death; NSVT, non-sustained ventricular tachycardia; LVOT, left ventricular outflow tract; NYHA, New York Heart Association; NT-pro ICD, implantable cardioverter-defibrillator; BNP, N-terminal pro-B-type natriuretic peptide; CK-MB, creatine kinase isomer-myoglobin; cTnI, cardiac troponin I; ACEi, angiotensin-converting enzyme inhibitors; ARB, angiotensin receptor blocker*.

+ *Expressed as mean ± standard deviation*.

# *Expressed as median (interquartile range)*.

$ *Postoperative drug therapy was not included*.

* *With statistically significant difference*.

Compared with the control group, end-systolic volume (ESV) was lower, while LVEF and LV myocardial mass were higher in HCM patients (all *P* < 0.001). Of all HCM patients, LVEF <50% and LV apical aneurysm were observed in seven (3.4%) and four patients (2.0%), respectively. The median maximal LVWT was 22.2 mm (interquartile range, 9.2) in patients with HCM. The global, minimal, and maximal native T1 and ECV values were significantly higher in patients with HCM (all *P* < 0.001). The median LGE/LV mass was 13.6% (21.3), with non-LGE in 15 patients (7.4%) and LGE/LV mass ≥15% in 88 patients (43.3%). According to the results of the control group, normal ranges of global native T1 mapping and ECV values in our institution were 1,180.4–1,299.6 ms and 21.4–29.4%, respectively. Increased global native T1 mapping (>1,299.6 ms) and increased global ECV (>29.4%) were found in 110 and 81 patients, respectively. The MRI parameters of HCM patients and control group are described in Table 2. Furthermore, phenotypes of HCM are presented in Table 3.

**Table 2:** Comparison of cardiac MRI data among healthy individuals and HCM patients.

**MRI parameters**	**Control group (*N* = 101)**	**HCM patients (*N* = 203)**	***P*-value**
EDV (ml)^#^	103.7 (31.1)	103.5 (40.1)	0.467
EDV/BSA (ml/m^2^)^#^	61.2 (12.4)	58.9 (18.7)	0.987
ESV (ml)^#^	29.4 (14.3)	23.9 (15.7)	<0.001^*^
ESV/BSA (ml/m^2^)^#^	17.8 (7.2)	13.7 (8.6)	<0.001^*^
LVEF (%)^#^	70.6 (6.5)	76.8 (12.3)	<0.001^*^
LVEF <50% (*N*, %)	0, 0%	7, 3.4%	0.059
Mass (g)^#^	92.4 (34.3)	184.4 (104.4)	<0.001^*^
Mass/BSA (g/m^2^)^#^	55.5 (16.0)	105.8 (56.2)	<0.001^*^
Maximal LVWT (mm)^#^	9.0 (2.0)	22.2 (9.2)	<0.001^*^
Maximal LVWT ≥30 mm (*N*, %)	0, 0%	40, 19.7%	<0.001^*^
LA diameter (mm)^#^	36.0 (5.5)	44.0 (6.0)	<0.001^*^
LGE/LV mass (%)^#^	0 (0, 0)	13.6 (21.3)	<0.001^*^
LGE/LV mass ≥15% (*N*, %)	0, 0%	88, 43.3%	<0.001^*^
Apical aneurysm (*N*, %)	0, 0%	4, 2.0%	0.156
Global native T1 (ms)^+^	1,240.0 ± 29.8	1,308.0 ± 55.5	<0.001^*^
Minimal native T1 (ms)^+^	1,185.2 ± 31.0	1,231.5 ± 70.9	<0.001^*^
Maximal native T1 (ms)^+^	1,320.1 ± 112.6	1,393.4 ± 140.4	<0.001^*^
Global ECV (%)^+^	25.4 ± 2.0	29.6 ± 6.0	<0.001^*^
Minimal ECV (%)^+^	22.5 ± 1.9	24.2 ± 3.5	<0.001^*^
Maximal ECV (%)^+^	29.2 ± 3.8	36.4 ± 9.6	<0.001^*^

*EDV, end diastolic volume; BSA, body surface area; ESV, end systolic volume; LVEF, left ventricular ejection fraction; LGE, late gadolinium enhancement; ECV, extracellular volume fraction; HCM, hypertrophic cardiomyopathy*.

+ *Expressed as mean ± standard deviation*.

# *Expressed as median (interquartile range*).

* *statistically significant difference*.

**Table 3:** Phenotype of HCM.

**Phenotype**	**Number of patients**
Focal basal septum HCM	67
Diffuse septum HCM	85
Concentric and diffuse HCM	6
Burned out phase HCM	4
Midventricular HCM	5
Apical HCM	27
Focal midseptum HCM	9

*HCM, hypertrophic cardiomyopathy*.

### Major SCD Risk Factors and Cardiac MRI Parameters in Two Guidelines

The 5-year SCD probability derived from the 2014 ESC guidelines for all HCM patients was 2.25% (1.73), in which 32 patients (15.8%) were classified as high risk. A total of 117 patients (57.6%) were ranked as high risk by the enhanced ACC/AHA guidelines. Differences between patients with low and high SCD risk assessed by the 2014 ESC guidelines and enhanced ACC/AHA guidelines are described in Supplementary Table 1.

The global native T1 and ECV showed a significant correlation (*r* = 0.307, *P* = 0.002 and *r* = 0.495, *P* < 0.001, respectively) in both the control group and HCM patients. In the HCM patients, the LGE/LV mass showed a significant correlation with global native T1 (*r* = 0.448, *P* < 0.001) and global ECV (*r* = 0.684, *P* < 0.001). Similarly, LGE/LV mass ≥15% also showed significant correlation with global native T1 (*r*_*s*_ = 0.538, *P* < 0.001) and ECV (*r*_*s*_ = 0.608, *P* < 0.001), respectively. Besides, the maximal LVWT, LA diameter, NSVT, and end-stage systolic dysfunction showed a significant correlation with global native T1 and ECV (*P* < 0.05; Table 4).

**Table 4:** Correlation between SCD risk factors and global T1 mapping parameters.

**Major risk factor**	**Global native T1**		**Global ECV**	
	***r* or *r_***s***_***	***P* value**	***r* or *r_***s***_***	***P*-value**
**2014 ESC guidelines major risk factors**				
Maximal LVWT	0.180	0.010^*^	0.163	0.020^*^
LA diameter	0.202	0.004^*^	0.219	0.002^*^
LVOT gradient pressure	−0.002	0.980	−0.151	0.032^*^
SCD family history	0.084	0.236	0.107	0.128
NSVT	0.212	0.002^*^	0.200	0.004^*^
Unexplained syncope	−0.026	0.714	0.047	0.507
Age	0.021	0.767	−0.123	0.081
**Enhanced ACC/AHA guidelines major risk factors**				
Maximal LVWT ≥30 mm	0.147	0.037^*^	0.205	0.003^*^
LGE/LV mass ≥15%	0.538	<0.001^*^	0.608	<0.001^*^
End-stage systolic dysfunction	0.248	<0.001^*^	0.293	<0.001^*^
LV apical aneurysm	0.051	0.466	0.135	0.055

*ECV, extracellular volume fraction; r, Pearson correlation coefficient; r_s_, Spearman correlation coefficient; ESC, European Society of Cardiology; LVWT, left ventricular wall thickness; LV, left ventricular; LA, left atrium; LVOT, left ventricular outflow tract; SCD, sudden cardiac death; NSVT, non-sustained ventricular tachycardia; ACC/AHA, American College of Cardiology/American Heart Association; LGE, late gadolinium enhancement*.

* *With statistically significant difference*.

### Cardiac MRI Between Two Guidelines

All 203 patients were grouped into three subgroups according to the 2014 ESC guidelines and the enhanced ACC/AHA guidelines. Subgroup 1 consisted of 86 patients (42.4%) and had lower risk according to both guidelines. Subgroup 2 comprised 85 patients (41.9%) and demonstrated conflicting results as evaluated by the two guidelines. The patients were classified as lower risk by the 2014 ESC guidelines but higher risk by the enhanced ACC/AHA guidelines. Subgroup 3 consisted of the remaining 32 patients (15.7%) evaluated as higher risk by both guidelines. Since none of the patients was considered to be high risk by the 2014 ESC guidelines and low risk by the enhanced ACC/AHA guidelines, the patients of subgroup 1 were patients ranked as lower risk by the enhanced ACC/AHA guidelines, and the patients of subgroups 2 and 3 were patients ranked as higher risk by the enhanced ACC/AHA guidelines.

All cardiac MRI parameters and T1 mapping values were significantly different among subgroups 1 to 3 except maximal native T1 and the presence of LV apical aneurysm (*P* < 0.05; Supplementary Table 2). Subgroup 3 had the highest EDV and LA diameter, while subgroup 1 had the least ESV, LV myocardial mass, maximal LVWT, and LGE/LV (all *P* < 0.05). All four cases with LV apical aneurysm were in subgroup 2. Subgroups 2 and 3 had higher global native T1 and ECV values than subgroup 1 did (all *P* < 0.05).

### Predictive Values of Native T1 Mapping and ECV

**Figure 2:**
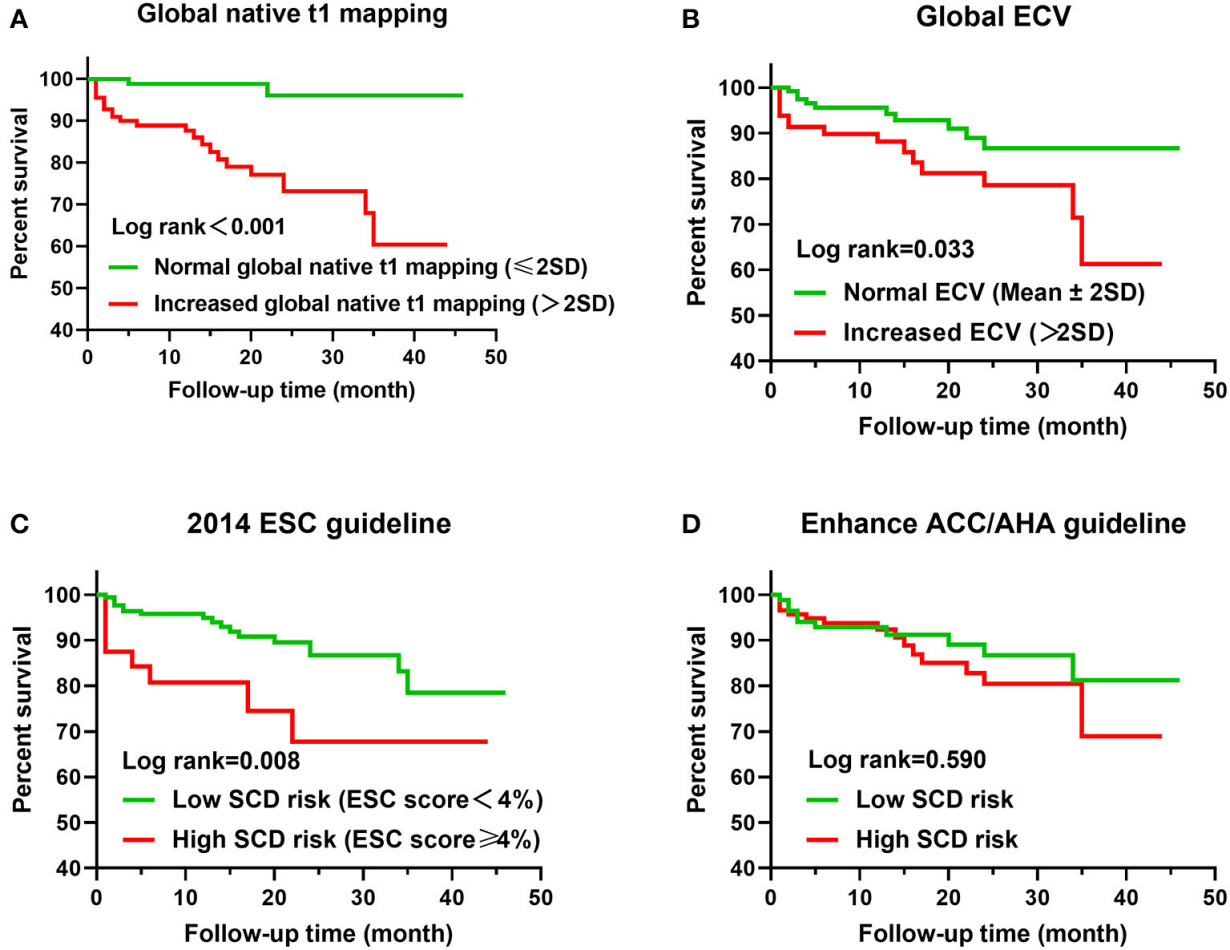
Kaplan–Meier curves for T1 mapping values, risk stratification of guidelines, and MACE. **(A)** Global native T1 mapping >2SD (60 ms) vs. ≤ 2SD. **(B)** Global ECV >2SD (4%) vs. ≤ 2SD. **(C)** ESC score <4 vs. ≥4%. **(D)** Low SCD risk assessed by enhanced ACC/AHA guidelines vs. high SCD risk.

**Figure 3:**
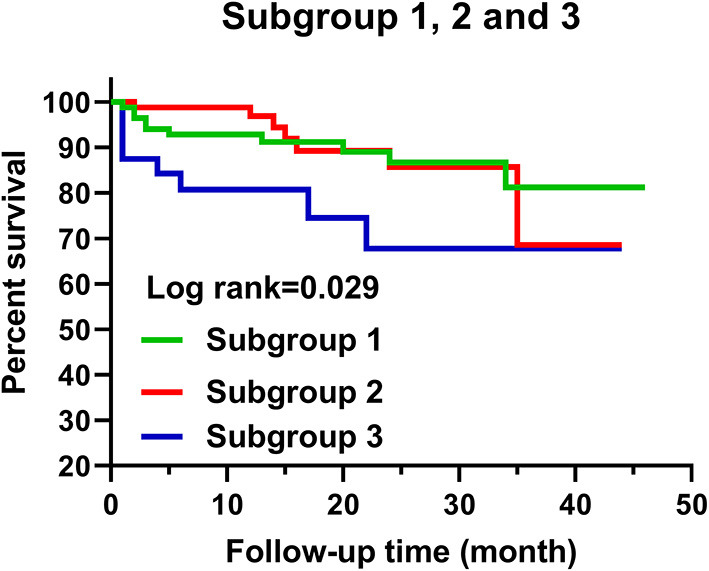
Kaplan–Meier curves for the classification of three subgroups and MACE.

During a median follow-up of 15 months (interquartile range, 19 months; range, 1–46 months), a total of 25 patients (12.3%) had MACE, including five cardiac death (three SCD), 10 placement of ICDs, two cardiac transplantation, two myocardial infarction, and six heart failure hospitalization. Placement of ICDs was due to III° atrial-ventricular block (four patients), sinus arrest (one patient), and NSVT (five patients). Median interval time between cardiac MRI and MACE was 6 months (interquartile range, 16.5 months; range, 1–35 months). In a total of 203 patients with HCM, Kaplan–Meier curves demonstrated that lower MACE-free survival was significantly associated with increased global native T1 mapping, increased global ECV, and high SCD risk assessed by the 2014 ESC guidelines (*P* < 0.001; Figure 2). However, SCD risk stratification assessed by the enhanced ACC/AHA guidelines was not associated with MACE-free survival. In addition, MACE-free survival was significantly different among the three subgroups (Figure 3).

**Figure 4:**
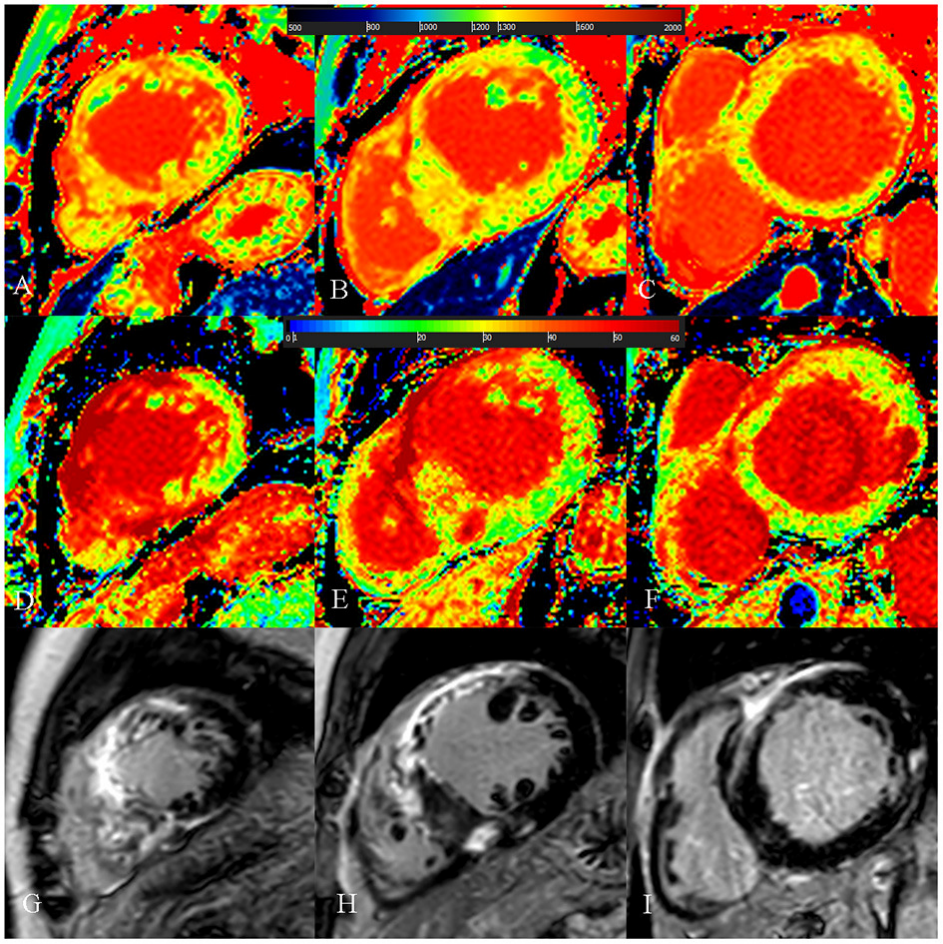
A 54-year-old male with diffuse septum hypertrophic cardiomyopathy. **(A**–**C)** Native T1 mapping of the apical, mid-, and basal portions of the left ventricular (LV) myocardium revealed higher global native T1 (1,359.67 ms). **(D**–**F)** Extracellular volume fraction (ECV) of the apical, mid-, and basal portions of the LV myocardium showed higher ECV (38.9%). **(G**–**I)** Late gadolinium enhancement (LGE) images of the apical, mid-, and basal portions of the LV myocardium showed multiple LGE. The maximal LV wall thickness was 24.3 mm, left atrial diameter was 55 mm, and the LV outflow tract gradient pressure was normal. He had non-sustained ventricular tachycardia but no family history of sudden cardiac death (SCD) and unexplained syncope. He had LGE/LV mass ≥15% (LGE/LV mass: 46.3%) and end-stage systolic dysfunction but no apical aneurysm. He was stratified as having high SCD risk under the 2014 European Society of Cardiology guidelines (5-year SCD probability: 5.93%) and enhanced American College of Cardiology/American Heart Association guidelines. He underwent the placement of ICD 6 months after cardiac MRI examinations. Higher global native T1 mapping and ECV values also indicated poor outcome.

Univariate Cox regression analysis showed that NSVT, LGE/LV mass, and end-stage systolic dysfunction were 7.901, 1.025, and 8.687 times higher in patients with MACE, respectively (*P* < 0.05; Table 5). In addition, global native T1 mapping, minimum native T1 mapping, global ECV, minimum ECV, and maximum ECV were 1.104–1.505 times higher in patients with MACE (*P* < 0.05). Furthermore, multivariate Cox regression analysis demonstrated that global native T1 mapping (hazard ratio (HR): 1.446; 95% confidence interval (CI): 1.195–1.749; *P* < 0.001) and NSVT (HR: 4.949; 95% CI: 2.033–12.047; *P* < 0.001) were independently associated with the incidence of MACE (Figure 4). It is worth noting that the presence of increased global native T1 mapping and ECV were excluded from the multivariate analysis due to their colinearity with global native T1 mapping and ECV values.

**Table 5:** Univariate and multivariate Cox regression analysis of major SCD risks and MRI parameters for the prediction of MACE in all patients with HCM.

	**Patients without MACE (*N* = 178)**	**Patients with MACE (*N* = 25)**	**HR (95% CI)**	***P*-value**
**Univariate cox regression analysis**				
**Major risk factors**				
Maximal LVWT (mm)	22.0 (9.3)	19.1 (8.8)	0.979 (0.911–1.053)	0.570
LA diameter (mm)	43.0 (6.0)	45.0 (8.5)	1.059 (0.998–1.123)	0.058
LVOT gradient pressure (mmHg)	31.5 (52.3)	15.0 (47.5)	0.995 (0.983–1.008)	0.458
SCD family history (*N*, %)	12, 6.7%	1, 4.0%	0.809 (0.109–6.011)	0.835
NSVT (*N*, %)	4, 2.2%	7, 28.0%	7.901 (3.293–18.953)	<0.001^*^
Unexplained syncope (*N*, %)	25, 14.0%	3, 12.0%	0.888 (0.266–2.970)	0.848
Age (years)	53.3 ± 15.2	60.1 ± 11.6	1.030 (0.999–1.062)	0.059
ESC score (%)	2.27 (1.69)	1.70 (3.49)	1.122 (0.907–1.390)	0.289
High SCD risk by 2014 ESC guideline (*N*, %)	24, 13.5%	8, 32.0%	2.954 (1.271–6.862)	0.012^*^
Maximal LVWT ≥30 mm (*N*, %)	36, 20.2%	4, 16.0%	0.892 (0.305–2.604)	0.834
LGE/LV mass ≥15% (*N*, %)	75, 42.1%	13, 52.0%	1.694 (0.770–3.726)	0.190
LGE/LV mass (%)	13.3 (18.7)	16.3 (35.8)	1.025 (1.005–1.046)	0.015^*^
End-stage systolic dysfunction (*N*, %)	1, 0.6%	6, 24.0%	8.687 (3.449–21.878)	<0.001^*^
LV apical aneurysm (*N*, %)	3, 1.7%	1, 4.0%	3.387 (0.449–25.554)	0.237
High SCD risk by enhanced ACC/AHA guideline (*N*, %)	102, 57.3%	15, 60.0%	1.368 (0.611–3.061)	0.446
**Cardiac MRI parameters**^**$**^				
EDV (ml)^#^	104.5 (37.8)	100.8 (84.1)	1.009 (1.002–1.015)	0.006^*^
EDV/BSA (ml/m^2^)^#^	58.8 (18.3)	61.1 (34.7)	1.016 (1.006–1.025)	0.001^*^
ESV (ml)^#^	24.0 (14.8)	23.0 (66.0)	1.011 (1.006–1.016)	<0.001^*^
ESV/BSA (ml/m^2^)^#^	13.8 (8.2)	13.3 (33.96)	1.017 (1.009–1.026)	<0.001^*^
LVEF (%)^#^	76.9 (11.7)	75.9 (32.3)	0.957 (0.936–0.979)	<0.001^*^
MASS (g)^#^	188.6 (97.6)	158.5 (160.8)	1.001 (0.996–1.005)	0.833
MASS/BSA (g/m^2^)^#^	106.8 (54.0)	100.0 (68.8)	1.001 (0.992–1.010)	0.805
Global native T1 (ms)^+^	1,301.5 ± 53.1	1,356.2 ± 47.3	1.492 (1.259–1.769)	<0.001^*^
Increased global native T1 (*N*, %)	87, 48.9%	23, 92.0%	10.750 (2.533–45.623)	0.001^*^
Minimal native T1 (ms)^+^	1,225.2 ± 71.0	1,274.1 ± 52.3	1.505 (1.231–1.840)	<0.001^*^
Maximal native T1 (ms)^+^	1,388.4 ± 146.9	1,430.2 ± 71.0	1.046 (0.987–1.109)	0.126
Global ECV (%)^+^	29.0 ± 5.4	34.3 ± 8.0	1.150 (1.074–1.230)	<0.001^*^
Increased global ECV (*N*, %)	66, 37.1%	15, 60.0%	2.323 (1.043–5.174)	0.039^*^
Minimal ECV (%)^+^	23.9 ± 3.3	26.3 ± 3.8	1.290 (1.103–1.508)	0.001^*^
Maximal ECV (%)^+^	35.3 ± 8.6	43.7 ± 13.1	1.104 (1.051–1.160)	<0.001^*^
**Multivariate cox regression analysis**				
Global native T1	–	–	1.446 (1.195–1.749)	<0.001^*^
NSVT	–	–	4.949 (2.033–12.047)	<0.001^*^

*SCD, sudden cardiac death; MACE, major adverse cardiovascular events; HCM, hypertrophic cardiomyopathy; HR, hazard ratio; CI, confidence interval; LVWT, left ventricular wall thickness; LA, left atrial; LVOT, left ventricular outflow tract; NSVT, non-sustained ventricular tachycardia; ESC, European Society of Cardiology; LGE, late gadolinium enhancement; LV, left ventricular; ACC/AHA, American College of Cardiology/American Heart Association; MRI, magnetic resonance imaging; ECV, extracellular volume fraction*.

+ *Expressed as mean ± standard deviation*.

# *Expressed as median (interquartile range*).

$ *Native T1 values were set as per SD (30 ms) increase; ECV values were set as per SD (2%) increase*.

* *statistically significant difference*.

**Figure 5:**
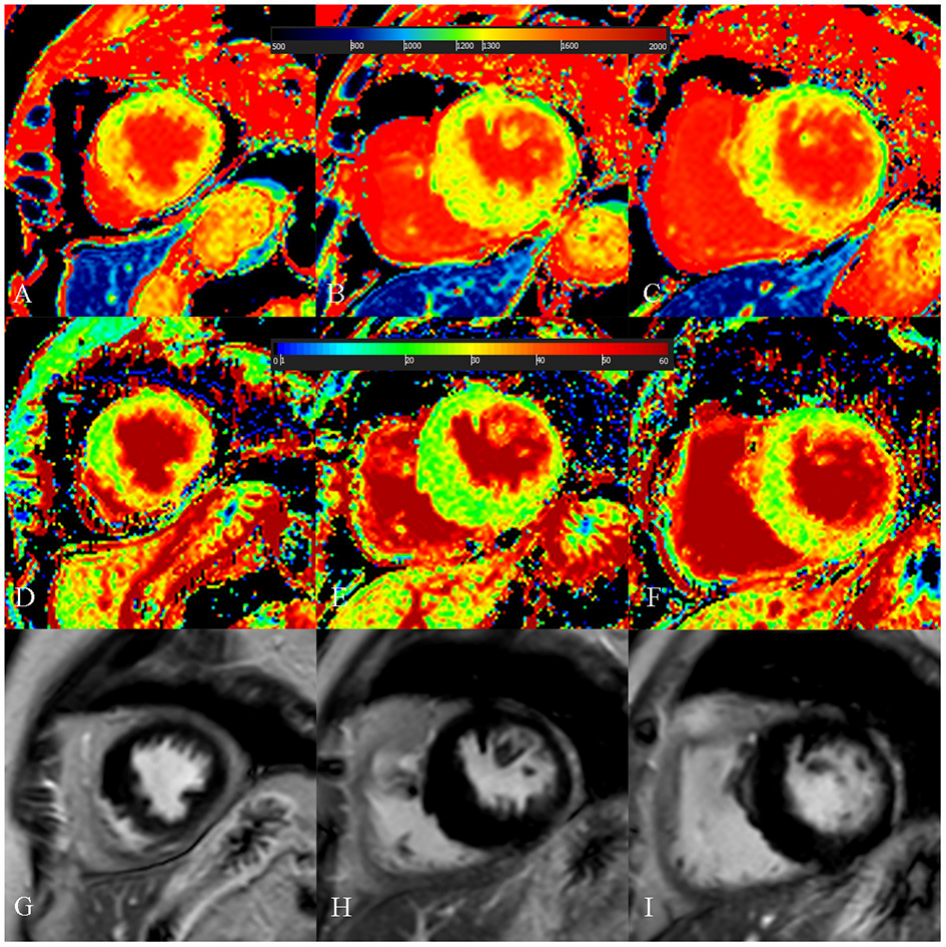
A 73-year-old female with focal midseptum hypertrophic cardiomyopathy. **(A**–**C)** Native T1 mapping of the apical, mid-, and basal portions of the left ventricular (LV) myocardium showed increased global native T1 (1,335.03 ms). **(D**–**F)** Extracellular volume fraction (ECV) of the apical, mid-, and basal portions of the LV myocardium showed normal global ECV (26.9%). **(G**–**I)** Late gadolinium enhancement (LGE) images of the apical, mid-, and basal portions of the LV myocardium showed no LGE. The maximal LV wall thickness was 19.8 mm, left atrial diameter was 40 mm, and the LV outflow tract gradient pressure was 95 mmHg. She had no family history of sudden cardiac death (SCD), non-sustained ventricular tachycardia, unexplained syncope, LGE/LV mass ≥15%, end-stage systolic dysfunction, and apical aneurysm. She was stratified as having low SCD risk under the 2014 European Society of Cardiology guidelines (5-year SCD probability: 1.70%) and enhanced American College of Cardiology/American Heart Association guidelines. However, she underwent cardiac-caused death 26 months after cardiac MRI examination. The elevated global native T1 mapping values could help indicate poor outcome in patients with low risk according to the two guidelines.

**Figure 6:**
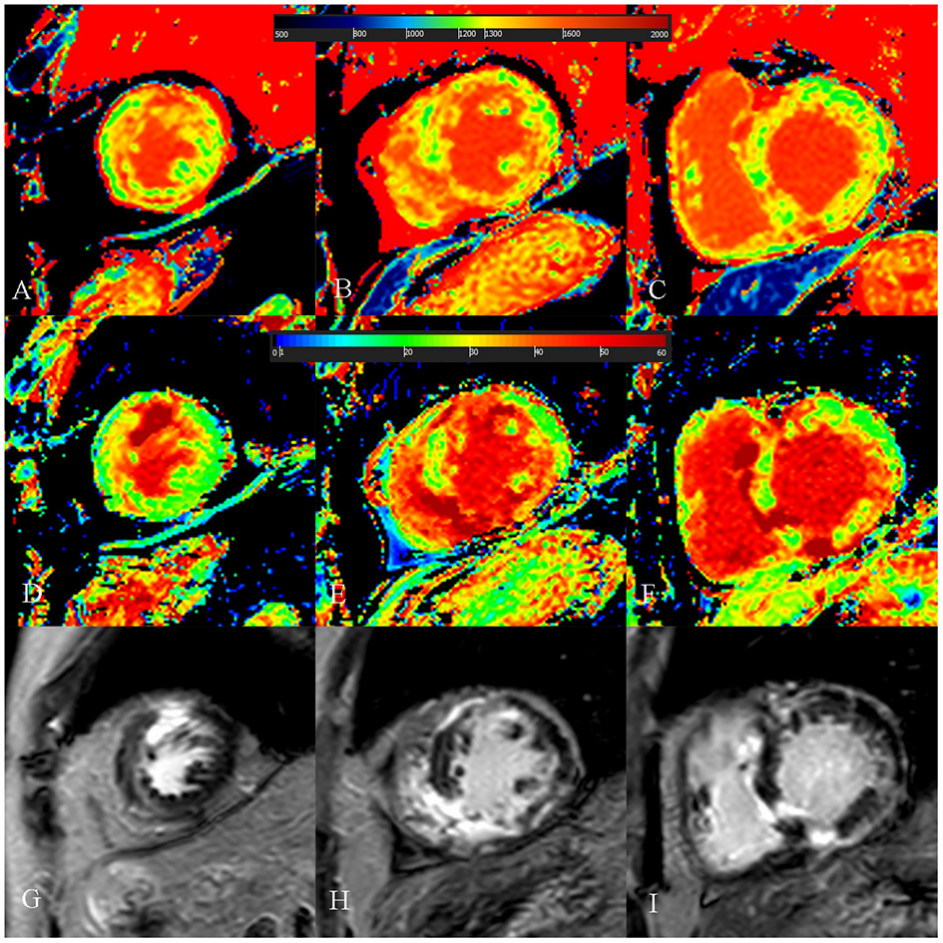
A 52-year-old male with burned out phase hypertrophic cardiomyopathy. **(A**–**C)** Native T1 mapping of the apical, mid-, and basal portions of the left ventricular (LV) myocardium revealed higher global native T1 (1,380.7 ms). **(D**–**F)** Extracellular volume fraction (ECV) of the apical, mid-, and basal portions of the LV myocardium showed higher global ECV (32.8%). **(G**–**I)** Late gadolinium enhancement (LGE) images of the apical, mid-, and basal portions of the LV myocardium showed multiple LGE. The maximal LV wall thickness was 13 mm, left atrial diameter was 53 mm, and the LV outflow tract gradient pressure was 2 mmHg. He had non-sustained ventricular tachycardia, but no family history of sudden cardiac death (SCD), and unexplained syncope. He had LGE/LV mass ≥15% (elevated LGE/LV mass: 56.2%) and end-stage systolic dysfunction but no apical aneurysm. He was stratified with low SCD risk under the 2014 European Society of Cardiology guidelines (5-year SCD probability: 3.39%) and high SCD risk under the enhanced American College of Cardiology/American Heart Association guidelines. He underwent cardiac transplantation 35 months after cardiac MRI examination. End-stage systolic dysfunction strongly indicated poor outcome in this patient. However, increased global native T1 mapping and ECV values could also suggest that the patient was likely to be at high risk of SCD and needs further treatment.

To further investigate the extra values of native T1 mapping and ECV in the prediction of MACE, Cox regression analysis were performed in three subgroups of patients. Ten patients in subgroup 1 (11.6%), seven patients in subgroup 2 (8.2%), and eight patients in subgroup 3 (25%) underwent MACE. In subgroup 1, univariate Cox regression analysis showed that global and minimum native T1 mapping and ECV, and age were 1.110–1.785 times higher in patients with MACE (*P* < 0.05; Table 6). Multivariate Cox regression analysis demonstrated that global native T1 mapping (HR: 1.532; 95% CI: 1.221–1.922; *P* < 0.001) was independently associated with the occurrence of MACE (Figure 5). In subgroup 2, univariate regression analysis showed that global native T1 mapping and end-stage systolic dysfunction were 1.518 and 6.472 times higher in patients with MACE (*P* < 0.05; Table 7). However, multivariate regression analysis revealed that only end-stage systolic dysfunction was independently associated with MACE (HR: 7.942; 95% CI: 1.322–47.707; *P* = 0.023; Figure 6). In subgroup 3, univariate Cox regression analysis showed that several parameters were significantly associated with MACE, but multivariate regression revealed that only NSVT was independently associated with MACE (HR: 9.779; 95% CI: 1.953–48.964; *P* = 0.006; Table 8).

**Table 6:** Univariate and multivariate Cox regression analysis of T1 mapping values for the prediction of MACE in subgroup 1.

	**Patients without MACE (*N* = 76)**	**Patients with MACE (*N* = 10)**	**HR (95% CI)**	***P*-value**
**Univariate cox regression analysis**				
**Major SCD risk factor**				
Maximal LVWT (mm)^#^	19.0 (6.1)	19.1 (3.5)	0.977 (0.825–1.157)	0.977
LA diameter (mm)^#^	43.0 (6.0)	42.5 (4.5)	1.024 (0.905–1.158)	0.711
LVOT gradient pressure (mmHg)^#^	30.0 (47.3)	47.5 (74.0)	1.011 (0.993–1.029)	0.238
Age (years)^+^	55.3 ± 14.3	69.0 ± 7.7	1.110 (1.030–1.196)	0.006^*^
LGE/LV mass (%)^#^	8.0 (6.4)	7.6 (7.8)	0.953 (0.821–1.107)	0.529
**Cardiac MRI mapping parameters**^**$**^				
Global native T1 (ms)^+^	1,277.9 ± 45.2	1,341.2 ± 39.6	1.532 (1.221–1.922)	<0.001^*^
Increased global native T1 (*N*, %)	18, 23.7%	9, 90.0%	19.812 (2.508–156.495)	0.005^*^
Minimal native T1 (ms)^+^	1,208.4 ± 72.9	1,281.4 ± 54.6	1.635 (1.259–2.125)	<0.001^*^
Maximal native T1 (ms)^+^	1,368.3 ± 172.8	1,407.9 ± 47.8	1.033 (0.951–1.121)	0.445
Global ECV (%)^+^	26.0 ± 2.9	28.8 ± 1.7	1.785 (1.146–2.780)	0.010^*^
Increased global ECV (*N*, %)	9, 11.8%	3, 30.0%	2.314 (0.590–9.075)	0.229
Minimal ECV (%)^+^	22.4 ± 3.9	24.8 ± 2.2	1.540 (1.056–2.244)	0.025^*^
Maximal ECV (%)^+^	31.4 ± 5.7	34.3 ± 4.8	1.100 (0.946–1.280)	0.215
**Multivariate cox regression analysis**				
Global native T1	–	–	1.532 (1.221–1.922)	<0.001^*^

*SCD, sudden cardiac death; MACE, major adverse cardiovascular events; HR, hazard ratio; CI, confidence interval; LVWT, left ventricular wall thickness; LA, left atrial; LVOT, left ventricular outflow tract; LGE, late gadolinium enhancement; LV, left ventricular; ECV, extracellular volume fraction*.

+ *Expressed as mean ± standard deviation*.

# *Expressed as median (interquartile range*).

$ *Native T1 values were set as per SD (30 ms) increase; ECV values were set as per SD (2%) increase*.

* *statistically significant difference*.

**Table 7:** Univariate and multivariate Cox regression analysis of T1 mapping values for the prediction of MACE in subgroup 2.

	**Patients without MACE (*N* = 78)**	**Patients with MACE (*N* = 7)**	**HR (95% CI)**	***P*-value**
**Univariate cox regression analysis**				
**Major SCD risk factor**				
Maximal LVWT (mm)^#^	24.3 (9.5)	22.3 (14.1)	1.003 (0.891–1.129)	0.957
LA diameter (mm)^#^	43.0 (6.0)	48.0 (11.0)	1.104 (0.955–1.275)	0.182
LVOT gradient pressure (mmHg)^#^	24.0 (50.5)	15.0 (45.0)	0.991 (0.966–1.017)	0.485
SCD family history (*N*, %)	7, 9.0%	0, 0%	–	–
NSVT (*N*, %)	1, 1.3%	1, 14.3%	2.249 (0.250–20.257)	0.470
Unexplained syncope (*N*, %)	13, 16.7%	0, 0%	–	–
Age (years)^+^	54.8 ± 15.2	57.1 ± 12.0	0.999 (0.946–1.055)	0.972
Maximal LVWT ≥30 mm (*N*, %)	26, 33.3%	2, 28.6%	0.948 (0.182–4.937)	0.949
LGE/LV mass ≥15% (*N*, %)	59, 75.6%	6, 85.7%	1.670 (0.201–13.884)	0.635
End-stage systolic dysfunction (*N*, %)	1, 1.3%	3, 42.9%	6.472 (1.397–29.976)	0.017^*^
LV apical aneurysm (*N*, %)	3, 3.8%	1, 14.3%	7.318 (0.809–66.202)	0.077
**Cardiac MRI mapping parameters**^**$**^				
Global native T1 (ms)^+^	1,319.1 ± 54.7	1,368.0 ± 66.5	1.518 (1.048–2.199)	0.027^*^
Increased global native T1 (*N*, %)	52, 66.7%	7, 100.0%	33.058 (0.014–77,171.798)	0.377
Minimal native T1 (ms)^+^	1,236.1 ± 70.5	1,275.8 ± 65.9	1.373 (0.852–2.212)	0.193
Maximal native T1 (ms)^+^	1,412.0 ± 136.8	1,451.6 ± 91.8	1.074 (0.926–1.245)	0.348
Global ECV (%)^+^	31.2 ± 6.2	36.9 ± 5.8	1.107 (0.975–1.257)	0.117
Increased global ECV (*N*, %)	42, 53.8%	6, 85.7%	3.629 (0.432–30.514)	0.235
Minimal ECV (%)^+^	24.8 ± 3.4	27.3 ± 4.5	1.186 (0.891–1.579)	0.243
Maximal ECV (%)^+^	38.7 ± 9.7	48.4 ± 9.6	1.087 (0.995–1.188)	0.064
**Multivariate cox regression analysis**				
End-stage systolic dysfunction (*N*, %)	–	–	7.942 (1.322–47.707)	0.023^*^

*SCD, sudden cardiac death; MACE, major adverse cardiovascular events; HR, hazard ratio; CI, confidence interval; ECV, extracellular volume fraction*.

+ *Expressed as mean ± standard deviation*.

# *Expressed as median (interquartile range*).

$ *Native T1 values were set as per SD (30 ms) increase; ECV values were set as per SD (2%) increase*.

* *statistically significant difference*.

**Table 8:** Univariate and multivariate cox regression analysis of T1 mapping values for the prediction of MACE in subgroup 3.

	**Patients without MACE (*N* = 24)**	**Patients with MACE (*N* = 8)**	**HR (95% CI)**	***P*-value**
**Univariate cox regression analysis**				
**Major SCD risk factor**				
Maximal LVWT (mm)^#^	26.0 (6.9)	17.9 (8.3)	0.861 (0.735–1.009)	0.064
LA diameter (mm)^#^	47.0 (9.0)	50.0 (8.5)	1.011 (0.924–1.107)	0.811
LVOT gradient pressure (mmHg)^#^	44.0 (57.0)	2.0 (25.9)	0.954 (0.917–0.992)	0.019^*^
SCD family history (*N*, %)	6, 22.2%	0, 0%	0.629 (0.077–5.121)	0.665
NSVT (*N*, %)	5, 18.5%	4, 80.0%	9.779 (1.953–48.964)	0.006^*^
Unexplained syncope (*N*, %)	13, 48.1%	2, 40.0%	0.630 (0.150–2.644)	0.528
Age (years)	43.0 ± 12.9	52.8 ± 9.3	1.046 (0.990–1.105)	0.106
Maximal LVWT ≥30 mm (*N*, %)	11, 40.7%	1, 20.0%	0.381 (0.098–2.426)	0.381
LGE/LV mass ≥15% (*N*, %)	19, 70.4%	4, 80.0%	2.981 (0.366–24.273)	0.307
End-stage systolic dysfunction (*N*, %)	1, 3.7%	2, 40.0%	13.104 (2.546–67.440)	0.002^*^
**Cardiac MRI mapping parameters**^**$**^				
Global native T1 (ms)^+^	1,318.6 ± 43.2	1,358.3 ± 50.6	1.741 (1.038–2.921)	0.036^*^
Increased global native T1 (*N*, %)	17, 70.8%	7, 87.5%	3.634 (0.433–30.517)	0.235
Minimal native T1 (ms)^+^	1,243.0 ± 56.7	1,269.8 ± 41.6	1.308 (0.887–1.930)	0.175
Maximal native T1 (ms)^+^	1,375.6 ± 51.4	1,435.5 ± 83.9	1.409 (1.058–1.875)	0.019^*^
Global ECV (%)^+^	31.0 ± 3.9	38.9 ± 10.6	1.236 (1.059–1.442)	0.007^*^
Increased global ECV (*N*, %)	15, 62.5%	6, 75.0%	1.728 (0.347–8.596)	0.504
Minimal ECV (%)^+^	25.4 ± 2.7	27.2 ± 4.6	1.333 (0.875–2.030)	0.180
Maximal ECV (%)^+^	37.0 ± 7.5	51.3 ± 16.4	1.150 (1.044–1.267)	0.005^*^
**Multivariate cox regression analysis**				
NSVT	–	–	9.779 (1.953–48.964)	0.006^*^

*SCD, sudden cardiac death; MACE, major adverse cardiovascular events; HR, hazard ratio; CI, confidence interval; ECV, extracellular volume fraction*.

+ *Expressed as mean ± standard deviation*.

# *Expressed as median (interquartile range*).

$ *Native T1 values were set as per SD (30 ms) increase; ECV values were set as per SD (2%) increase*.

* *statistically significant difference*.

## Discussion

Our study has demonstrated several important findings: (1) global native T1 mapping and NSVT are independent risk factors associated with MACE; (2) in patients stratified as low SCD risk by the two guidelines, global native T1 mapping has incremental predictive values over the two guidelines and is independently associated with poor outcomes; and (3) in patients with conflicting results assessed by the two guidelines, although end-stage systolic dysfunction is a powerful predictor, global native T1 mapping could also be helpful to indicate the poor outcome.

The 2014 ESC guidelines and enhanced ACC/AHA guidelines are currently the most two common guidelines to judge the risk for SCD and select patients for ICDs. However, there is no consensus reached as to which guideline is better and the evaluation of SCD risk is still challenging. In a study of 3,703 HCM patients, O'Mahony et al. have evidenced that the 2014 ESC guidelines demonstrated good discrimination between patients who should and should not receive ICDs (24). However, another large study involving 2,094 HCM patients conducted by Maron et al. revealed that the enhanced ACC/AHA guidelines had a higher sensitivity (79–93%) but lower specificity (76–80%) in discriminating patients with and without SCD events, whereas the 2014 ESC guidelines had a much lower sensitivity (47–69%) but slightly higher specificity (79–82%) (9). Liu et al. have demonstrated that the enhanced ACC/AHA guidelines assisted in better predicting for SCD risk stratification than the 2014 ESC guidelines did in 1,369 HCM patients (25). These uncertainties urge the need to introduce novel biomarkers to supplement the current guidelines (26). In this study, during a median follow-up of 15 months, we revealed that the global native T1 mapping was an independent predictor of MACE in HCM. It was not confounded by the traditional imaging risk factors, including maximal LVWT, LA diameter, and LGE/LV mass. Our findings are important because the global native T1 mapping might have the potential to be a supplement to the current guidelines and a new biomarker for SCD risk stratification. It could assist in the clinical decision making to prevent adverse outcomes in HCM, especially when the stratification and management could not be determined by the current guidelines.

To deeply excavate the incremental values of T1 mapping values over the current guidelines, we analyzed these parameters in the three subgroups stratified according to the two guidelines. It is noteworthy that we discover the strong independent association between native T1 mapping and the incidence of 10 MACE in 86 patients (11.6%, subgroup 1) assessed as low SCD risk according to the two guidelines. Choi et al. have also reported that seven SCD events happened in 615 patients with HCM in low-risk group assessed by the 2014 ESC guidelines (27). They suggested that the 2014 ESC guidelines might not be suitable in Asian patients and would unprotect the low-risk patients. Compared with their study, our results emphasize the extra values of the global native T1 mapping over the current two guidelines in the prediction of adverse outcomes. In this subgroup of patients who are not likely to receive the advanced therapies according to the current guidelines, increased global native T1 mapping could be helpful in the prediction of poor outcomes and the identification of patients who might benefit from ICDs. On the other hand, native T1 mapping and ECV values were not included in the multivariate analysis in patients of groups 2 and 3 due to the much stronger impact of end-stage systolic dysfunction and NSVT on the outcomes. However, these values were associated with the prediction of adverse outcomes in the univariate analysis and might be a supplement in the clinical decision when the traditional risk factors are not present.

There were limited studies with regard to the relationship between T1 mapping and ECV with the adverse outcomes in HCM patients (3). Li et al. have reported that ECV was a strong biomarker in predicting the adverse outcomes in 263 HCM patients during a mean follow-up of 28.3 months (23). Xu et al. found that both native T1 mapping and ECV values were associated with SCD in 258 patients with HCM without the presence of LGE and LVOT obstruction (14). Two baseline studies of small sample size also revealed the relationship between ECV and SCD risks (28, 29). Compared with the previous few studies, our results have added new evidence regarding the prognostic significance of global native T1 mapping and ECV in the evaluation of HCM.

Similar to our study, the importance of native T1 mapping values on the prognosis has been revealed by studies about other cardiovascular diseases. In two studies of non-ischemic dilated cardiomyopathy, native T1 mapping was an independent predictor for MACE-related endpoints (30, 31). In a meta-analysis by Pan et al., native T1 mapping had the similar sensitivity and specificity with ECV in the prediction of prognosis in cardiac amyloidosis (32). We speculate that some explanations might be responsible for the increased native T1 mapping in HCM. First, this quantitative parameter is prone to T2 decay and more sensitive to the change of water content within myocardium than ECV (33). The myocardial edema can be resulted by microvascular ischemia which is a commonplace in HCM (34). In addition, chronic heart failure is also characterized by myocardial edema (35, 36). This is particularly important in our study because quite a few patients are over 60 years and more related with the adverse outcome caused by heart failure (37). Alternatively, native T1 mapping is a robust biomarker indicating the pathologic hypertrophic remodeling in HCM (38, 39). The myocardial tissue remodeling including cardiomyocyte disarray is also a risk factor for malignant arrhythmia (15, 32, 40). Furthermore, native T1 mapping is important for the detection of focal and diffuse fibrosis without the administration of contrast (14). It is particularly useful when the diffuse fibrosis is negative and undetectable on LGE images.

## Limitations

Our study had some limitations. Firstly, this study was performed in a single center where T1 mapping sequences were scanned on a 3.0-T MRI scanner. Future study was required to validate whether our results could be used in other centers. Secondly, the occurrence of MACE rather than SCD was considered the primary endpoint in our study. The prevalence of SCD and placement of ICD is rare in our population, and this would cause a big problem of underestimation or overestimation in the statistical power if we set SCD as the only primary endpoint. Moreover, MACE such as myocardial infarction and acute heart failure would put patients at high risk of SCD (1, 9). SCD-free survival due to the timely rescue should not exclude these patients from the high-risk stratification. Thirdly, our follow-up period is relatively short and the sample size of three subgroups is small. Future studies should investigate the predictive ability of T1 mapping values in larger samples during the long-term follow-up. Fourthly, genetic tests were not performed in our study. This would preclude the enrollment of genotype-positive but hypertrophy-negative patients. In addition, the influence of various genotypes on the endpoints has not been analyzed in our study. However, as an update to the 2011 ACC/AHA guidelines, the enhanced ACC/AHA guidelines have removed genetic mutations as a high-risk factor (5, 9). Finally, the proportion of patients with LGE/LV mass ≥15% is relatively high (88, 43.3%). This might be the reason that the enhanced ACC/AHA guideline was not associated with MACE-free survival. However, the new guidelines detected more patients (15 patients) with MACE than the 2014 ESC guidelines did (eight patients). Therefore, our results should be cautiously interpreted in generalized HCM patients.

## Conclusion

Cardiac MRI T1 mapping, especially global native T1 mapping, could provide incremental values and serve as potential supplements to the current guidelines in the evaluation of high MACE risk and guide advanced therapies.

## Data Availability Statement

The datasets presented in this article are not readily available because our dataset is only available to our research team. Requests to access the datasets should be directed to Fuhua Yan, yfh11655@rjh.com.cn.

## Ethics Statement

The studies involving human participants were reviewed and approved by Ruijin Hospital Ethics Committee Shanghai JiaoTong University School of Medicine. Written informed consent to participate in this study was provided by the participants' legal guardian/next of kin.

## Author Contributions

LQ, JM, and CC drafted the manuscript, figures, and tables. LZ, SG, and MZ helped for figure and case preparation. FY and WY made a careful review of the manuscript. All authors contributed to this study by conceiving and designing the study, performing the data collection and the statistical analysis or assisting in data interpretation, and approved the submitted version.

## Conflict of Interest

The authors declare that the research was conducted in the absence of any commercial or financial relationships that could be construed as a potential conflict of interest.

## Abbreviations

HCM: hypertrophic cardiomyopathy
SCD: sudden cardiac death
ICD: implantable cardioverter-defibrillator
ESC: European Society of Cardiology
ACC/AHA: American College of Cardiology/American Heart Association
LGE: late gadolinium enhancement
LV: left ventricular
ECV: extracellular volume fraction
LVWT: left ventricular wall thickness
LVEF: left ventricular ejection fraction
LVOT: left ventricular outflow tract
NSVT: non-sustained ventricular tachycardia
MACE: major adverse cardiovascular events
HR: hazard ratio
CI: confidence interval.
