# Supplementary material for: Incremental Values of T1 Mapping in the Prediction of Sudden Cardiac Death Risk in Hypertrophic Cardiomyopathy: A Comparison With Two Guidelines
Source: Front Cardiovasc Med. 2021 Jun 8;8:661673. doi: 10.3389/fcvm.2021.661673 (PMC8217449; doi:10.3389/fcvm.2021.661673)
Supplement: Supplementary file 1 [file Data_Sheet_1.DOCX]

Supplementary S1

Differentiation of hypertensive heart disease (HHD) and hypertrophic cardiomyopathy (HCM) is according to the following standards: (1) HHD has reduced left ventricular ejection fraction, increased LV cavity size; concentric hypertrophy is the most common phenotype in HHD, and other phenotype is very rare; left ventricular outflow obstruction is rare in HHD; treatment with antihypertensive drugs leading to the reduced wall thickness favors the diagnosis of HHD; (2) HCM has supernormal ejection fraction, reduced LV cavity size; asymmetrical LV wall thickness is consistent with HCM; LV wall thickness of 18 mm or greater and mitral valve systolic anterior motion favors the diagnosis of HCM; late gadolinium enhancement is common at the right ventricular septal insertion sites and in those walls with the greatest hypertrophy in HCM (1-4). Patients were excluded from our study when it was difficult to differentiate between the two diseases.

Supplementary S2

Native T1 mapping: coil: 6-channel body coil; NSA:1; Trigger delay: longest; breathhold mode: expiration; cardiac device: ECG; R-R window: 10-20; Recon voxel size: 1.17×1.17mm; fold-over suppression: oversampling, 30×30mm; fold-over direction: RL; reconstruction matrix: 256; SENSE: yes; P reduction: 2; k-t acceleration: no; slice scan order: interleaved; planalign: yes; contrast enhancement: balanced; acquisition mode: Cartesian; fast imaging mode: TFE; ultrashort: yes; shot mode: single-shot; TFE startup echoes: 10; B-TFE startup mode: lin. sweep up; shot interval: shortest; profile order: linear; echoes: 1; partial echo: yes; partial echo factor: 0.85; shifted echo: no; halfscan: no; water-fat shift: 0.4; RF Shims: volume; Shim: volume; mDIXON: no; fat suppression: no; water suppression: no; TFE prepulse: invert (delay 350 ms); MTC: no; T2prep: no; Research prepulse: no; Diffusion mode: no; Transmit channels: both; SAR mode: high; B1 mode: default; SAR allow first level: yes; PNS mode: moderate; gradient mode: maximum; SofTone mode: no.

**Reference**

1. Baxi AJ, Restrepo CS, Vargas D, Marmol-Velez A, Ocazionez D, H M. Hypertrophic cardiomyopathy from A to Z genetics, pathophysiology, imaging, and management. *Radiographics*. (2016) 36:335-54. doi: 10.1148/rg.2016150137.

2. Eun Ju Chun, Sang Il Choi, Kwang Nam Jin, Hyon Joo Kwag, Young Jin Kim, Byoung Wook Choi, et al. Hypertrophic cardiomyopathy: assessment with MR imaging and multidetector CT. *Radiographics*. (2010) 30:1309-28. doi: 10.1148/rg.305095074.

3. Patel AR, Kramer CM. Role of cardiac magnetic resonance in the diagnosis and prognosis of nonischemic cardiomyopathy. *JACC Cardiovasc Imaging*. (2017) 10:1180-93. doi: 10.1016/j.jcmg.2017.08.005.

4. Maron MS, Rowin EJ, Maron BJ. How to Image Hypertrophic Cardiomyopathy. *Circ Cardiovasc Imaging*. (2017) 10. doi: 10.1161/CIRCIMAGING.116.005372.
