# Supplementary material for: Incremental Values of T1 Mapping in the Prediction of Sudden Cardiac Death Risk in Hypertrophic Cardiomyopathy: A Comparison With Two Guidelines
Source: Front Cardiovasc Med. 2021 Jun 8;8:661673. doi: 10.3389/fcvm.2021.661673 (PMC8217449; doi:10.3389/fcvm.2021.661673)
Supplement: Supplementary file 2 [file Data_Sheet_2.docx]

Table S1. Comparison of major SCD risk factors and cardiac MRI data among patients with lower and higher SDC assessed by 2014 ESC and Enhanced ACC/AHA Strategy

|  | 2014 ESC guidelines | | | Enhanced ACC/AHA guidelines | | |
| --- | --- | --- | --- | --- | --- | --- |
|  | Lower SCD risk (N=171) | Higher SCD risk (N=32) | P value | Lower SCD risk (N=86) | Higher SCD risk (N=117) | P value |
| Clinical characteristics |  |  |  |  |  |  |
| Age (years)^+^ | 56.0±14.6 | 44.5±12.8 | 0.006 | 56.9±14.4 | 52.1±15.1 | 0.023 |
| SCD family history (N, %) | 7, 4.1% | 6, 18.8% | 0.002 | 0, 0% | 13, 11.1% | 0.001 |
| Unexplained syncope (N, %) | 13, 7.6% | 15, 46.9% | ＜0.001 | 0, 0% | 28, 23.9% | ＜0.001 |
| NSVT (N, %) | 2, 1.2% | 9, 28.1% | ＜0.001 | 0, 0% | 11, 9.4% | 0.004 |
| Echocardiography |  |  |  |  |  |  |
| LOVT pressure gradient (mmHg)^#^ | 27.0 (50.0) | 41.5 (66.3) | 0.065 | 31.5 (49.3) | 30.0 (51.5) | 0.867 |
| MRI parameters |  |  |  |  |  |  |
| EDV (mL)^#^ | 101.8 (35.5) | 122.8 (55.5) | 0.001 | 100.8 (35.9) | 108.7 (46.1) | 0.068 |
| EDV/BSA (mL/m^2^)^#^ | 57.7 (17.2) | 72.6 (25.0) | ＜0.001 | 56.9 (16.3) | 61.4 (19.5) | 0.015 |
| ESV (mL)^#^ | 23.0 (14.6) | 28.5 (18.3) | 0.006 | 21.3 (11.4) | 27.0 (19.4) | ＜0.001 |
| ESV/BSA (mL/m^2^)^#^ | 13.2 (8.3) | 16.0 (10.5) | 0.006 | 11.8 (5.8) | 15.5 (10.3) | ＜0.001 |
| LVEF (%)^#^ | 76.9 (12.1) | 76.1 (12.9) | 0.249 | 79.0 (7.9) | 74.4 (13.1) | ＜0.001 |
| LVEF＜50% (N, %) | 4, 2.3% | 3, 9.4% | 0.046 | 0, 0% | 7, 6.0% | 0.021 |
| MASS (g)^#^ | 174.0 (100.1) | 234.5 (104.5) | 0.002 | 164.1 (81.3) | 202.9 (129.9) | 0.001 |
| MASS/BSA (g/m^2^)^#^ | 101.4 (52.8) | 123.4 (52.5) | 0.001 | 90.3 (40.3) | 118.2 (64.6) | ＜0.001 |
| Maximal LV wall thickness (mm)^#^ | 21.0 (8.8) | 24.8 (6.4) | 0.001 | 19.0 (5.5) | 24.6 (8.9) | ＜0.001 |
| Maximal LV wall thickness≥30mm (N, %) | 28, 16.4% | 12, 37.5% | 0.006 | 0, 0% | 40, 34.2% | ＜0.001 |
| LA diameter (mm)^#^ | 42.8 (6.0) | 47.9 (8.8) | ＜0.001 | 43.0 (6.0) | 44.0 (8.0) | 0.008 |
| LGE/LV mass^#^ | 12.6 (17.0) | 23.2 (27.1) | 0.002 | 8.0 (6.6) | 23.5 (20.8) | ＜0.001 |
| LGE/LV mass≥15% (N, %) | 65, 38.0% | 23, 71.9% | ＜0.001 | 0, 0% | 88, 75.2% | ＜0.001 |
| Apical aneurysm (N, %) | 4, 2.3% | 0, 0% | 0.383 | 0, 0% | 4, 3.4% | 0.084 |
| Global native T1 (msec)^+^ | 1304.1±56.1 | 1328.5±47.6 | 0.022 | 1285.3±48.8 | 1324.6±54.4 | ＜0.001 |
| Abnormal native T1 (N, %) | 85, 49.7% | 24, 75.0% | 0.009 | 27, 31.4% | 82, 70.1% | ＜0.001 |
| Minimal native T1 (msec)^+^ | 1228.0±73.3 | 1249.7±54.0 | 0.113 | 1216.9±74.5 | 1242.2±66.4 | 0.012 |
| Maximal native T1 (msec)^+^ | 1393.9±150.5 | 1390.6±65.1 | 0.901 | 1372.9±163.5 | 1408.5±119.2 | 0.074 |
| Global ECV (msec)^+^ | 29.0±5.6 | 33.0±7.0 | ＜0.001 | 26.3±2.9 | 32.0±6.5 | ＜0.001 |
| Abnormal global ECV | 60, 35.1% | 21, 65.6% | 0.001 | 12, 14.0% | 69, 59.0% | ＜0.001 |
| Minimal ECV (msec)^+^ | 23.9±3.4 | 25.9±3.3 | 0.002 | 22.7±2.9 | 25.3±3.5 | ＜0.001 |
| Maximal ECV (msec)^+^ | 35.6±9.0 | 40.6±11.9 | 0.006 | 31.7±5.6 | 39.8±10.5 | ＜0.001 |

ESC: European society of cardiology; ACC/AHA: American College of Cardiology/American Heart Association; EDV: end diastolic volume; BSA: body surface area; ESV: end systolic volume; EF: ejective fraction; LV: left ventricular; LGE: late gadolinium enhancement; ECV: extracellular volume fraction; HCM: hypertrophic cardiomyopathy.

^+^ expressed as mean±standard deviation

^#^ expressed as median (interquartile range)

Table S2. Comparison of cardiac MRI data among different classifications of 2014 ESC and enhanced ACC/AHA guidelines

| MRI parameters | Subgroup 1 (N=86) | Subgroup 2 (N=85) | Subgroup 3 (N=32) | P value |
| --- | --- | --- | --- | --- |
| EDV (mL)^#^ | 100.7 (35.9)^c^ | 104.3 (34.8)^c^ | 122.8 (55.5)^a,b^ | 0.003^*^ |
| EDV/BSA (mL/m^2^)^#^ | 56.9 (16.3)^c^ | 58.6 (18.1)^c^ | 72.6 (25.0)^a,b^ | ＜0.001^*^ |
| ESV (mL)^#^ | 21.3 (11.4)^b,c^ | 25.4 (19.8)^a^ | 28.5 (18.3)^a^ | ＜0.001^*^ |
| ESV/BSA (mL/m^2^)^#^ | 11.8 (5.8)^b,c^ | 15.5 (10.5)^a^ | 16.0 (10.5)^a^ | ＜0.001^*^ |
| LVEF (%)^#^ | 79.0 (7.9)^b^ | 74.3 (13.6)^a^ | 76.1 (12.9) | 0.001^*^ |
| LVEF＜50% (N, %) | 0, 0%^c^ | 4, 4.7% | 3, 9.4%^a^ | 0.032^*^ |
| MASS (g)^#^ | 164.1 (81.3)^b,c^ | 201.4 (137.4)^a^ | 234.5 (104.5)^a^ | 0.001^*^ |
| MASS/BSA (g/m^2^)^#^ | 90.3 (40.3)^b,c^ | 112.7 (67.8)^a^ | 123.4 (52.5)^a^ | ＜0.001^*^ |
| Maximal LVWT (mm)^#^ | 19.0 (5.5)^b,c^ | 24.0 (9.9)^a^ | 24.9 (6.4)^a^ | ＜0.001^*^ |
| Maximal LVWT≥30mm (N, %) | 0, 0%^b,c^ | 28, 32.9%^a^ | 12, 37.5%^a^ | ＜0.001^*^ |
| LA diameter (mm)^#^ | 43.0 (6.0)^c^ | 43.0 (6.0)^c^ | 47.5 (8.8)^a,b^ | ＜0.001^*^ |
| LGE/LV mass (%)^#^ | 8.0 (6.6)^b,c^ | 23.5 (20.0)^a^ | 23.2 (27.1)^a^ | ＜0.001^*^ |
| LGE/LV mass≥15% (N, %) | 0, 0%^b,c^ | 65, 76.5%^a^ | 23, 71.9%^a^ | ＜0.001^*^ |
| Apical aneurysm (N, %) | 0, 0% | 4, 4.7% | 0, 0% | 0.064 |
| Global native T1 (msec)^+^ | 1285.3±48.8^b,c^ | 1323.2±56.9^a^ | 1328.5±47.6^a^ | ＜0.001^*^ |
| Minimal native T1 (msec)^+^ | 1216.9±74.5^c^ | 1239.3±70.6 | 1249.7±54.0^a^ | 0.033^*^ |
| Maximal native T1 (msec)^+^ | 1372.9±163.5 | 1415.3±133.7 | 1390.6±65.1 | 0.141 |
| Global ECV (%)^+^ | 26.3±2.9^b,c^ | 31.7±6.3^a^ | 33.0±7.0^a^ | ＜0.001^*^ |
| Minimal ECV (%)^+^ | 22.7±2.9^b,c^ | 25.0±3.5^a^ | 25.9±3.3^a^ | ＜0.001^*^ |
| Maximal ECV (%)^+^ | 31.7±5.6^b,c^ | 39.5±10.0^a^ | 40.6±11.9^a^ | ＜0.001^*^ |

ESC: European society of cardiology; ACC/AHA: American College of Cardiology/American Heart Association; EDV: end diastolic volume; BSA: body surface area; ESV: end systolic volume; LVEF: left ventricular ejection fraction; LGE: late gadolinium enhancement; ECV: extracellular volume fraction; HCM: hypertrophic cardiomyopathy.

Subgroup 1: patients assessed as to be with lower risk by both two guidelines; Subgroup 2: patients classified as to be with lower risk by 2014 ESC guidelines but higher risk by enhanced ACC/AHA guidelines; Subgroup 3: patients evaluated as to be with higher risk by both guidelines.

a: different with subgroup1 with statistical significance; b: different with subgroup 2 with statistical significance; c: different with subgroup 3 with statistical significance;

^#^ expressed as median (interquartile range)

^+^ expressed as mean±standard deviation

^*^ with statistically significant difference
